# Molecular Mimicry and Uveitis

**DOI:** 10.3389/fimmu.2020.580636

**Published:** 2020-10-29

**Authors:** Gerhild Wildner, Maria Diedrichs-Möhring

**Affiliations:** Section of Immunobiology, Department of Ophthalmology, University Hospital, LMU Munich, München, Germany

**Keywords:** autoimmune disease, T cells, HLA, tolerance, nutritional antigens, microbiome, intraocular inflammation, pathogens

## Abstract

Molecular or antigenic mimicry is a term for the similarity of different antigens, which can be confused by the immune system. Antigen recognition by antibodies and T cell receptors is specific, but not restricted to a single antigen. Both types of receptors specifically recognize antigens and are expressed with a very high but still restricted variability compared to the number of different antigens they potentially could bind. T cell receptors only can bind to antigen peptides presented on certain self-MHC-molecules by screening only some amino acid side chains on both the presented peptides and the MHC molecule. The other amino acids of the peptide are not directly perceived by the T cell, offering the opportunity for a single T cell to recognize a variety of different antigens with the same receptor, which significantly increases the immune repertoire. The immune system is usually tolerant to autoantigens, especially to those of immune privileged sites, like the eye. Therefore, autoimmune diseases targeting these organs were hard to explain, unless a T cell is activated by an environmental peptide (e.g. pathogen) that is similar, but not necessarily identical with an autoantigen. Here we describe antigenic mimicry of retinal autoantigens with a variety of non-ocular antigens resulting in the induction of intraocular inflammation. T cells that are activated by mimotopes outside of the eye can pass the blood-retina barrier and enter ocular tissues. When reactivated in the eye by crossreaction with autoantigens they induce uveitis by recruiting inflammatory cells.

## Introduction

**Figure 1:**
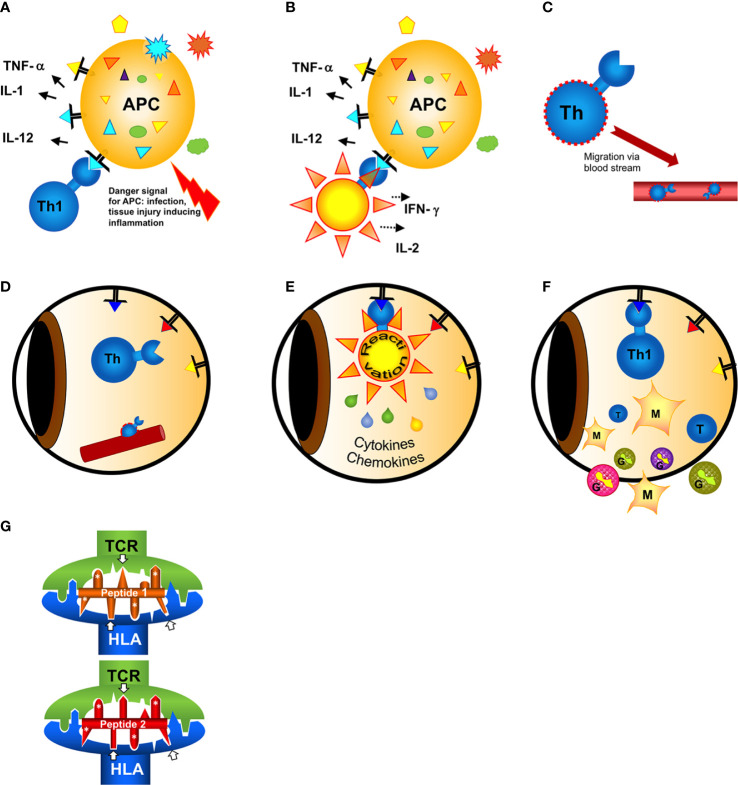
Hypothesis of the induction of a T cell mimicry response leading to ocular autoimmunity. **(A)** Outside of the eye in the body (gut)?: A naive T cell has first contact with a non-eye-related antigen. In case of a concomitant danger signal (injury of the tissue, infection) innate antigen-presenting cells (APCs) are activated to induce effector T cell responses. **(B)** The antigen recognized by the T cell can either be from a pathogen [from infection or a food antigen, see **(A)**]. If the T cell recognizes harmless food antigen and erroneously receives concomitant activation signals from the APC alerted by danger signals the T cell gets activated and differentiates into a T helper (e.g. here: Th1) cell. **(C)** Activated T cells migrate in the blood circulation. In search of their antigen they can leave the blood vessels and enter even immune privileged organs like the eye. **(D)** The triangles represent retinal autoantigen peptides presented on MHC class II. The precise signals of attracting a T cell to a certain organ at a certain site (blood vessel) are still unknown. **(E)** When the T cell has screened the eye and found an antigen binding to its receptor, it gets reactivated and secretes cytokines and chemokines to recruit inflammatory cells. The intraocular antigen is different to the antigen of the original T cell priming outside of the eye. **(F)** Innate cells (monocytes/macrophages and/or granulocytes) recruited from the circulation are causing inflammation and tissue destruction resulting in uveitis. **(G)** Two different peptides presented and recognized by the same HLA molecule and T cell receptor (TCR), despite their restricted homologies. Only the core sequence of 8 amino acids presented in the groove is shown. Identical amino acid side chains are marked by white asterisks, the arrows point to similar side chains representing different amino acids also anchoring to the presenting HLA-molecule or being bound by the TCR.

The inner eye as an immune privileged site is rarely affected by inflammation. Autoimmune uveitis with a frequency of 0.2% is an uncommon autoimmune diseases. The immune privilege is usually maintained by a number of mechanisms including anterior chamber-associated immune deviation, protective surfaces of intraocular cells, suppressive factors and the blood-retina-barrier (BRB) (1). The latter is impermeable for large molecules such as antibodies and non-activated lymphocytes, sequestering unique intraocular autoantigens from the immune system (2). Therefore, autoimmunity to intraocular antigens was initially hard to explain. Only previously activated lymphocytes are able to pass tissue barriers to screen tissues for potential hidden pathogens and tumor cells. An activation of lymphocytes to retinal autoantigens is only possible in the case of ocular trauma destroying the protective vascular barriers, the premise for the pathogenesis of sympathetic ophthalmia (3). In this condition injury to one eye allows local activation of T cells, which are then enabled to enter the partner eye to initiate a destructive autoimmune response. This is a very rare event. Usually intraocular inflammation occurs without any injury to the eye. Therefore, autoimmunity to intraocular antigens must be initiated by activated T cells, which had their primary antigen contact outside of the eye. This antigen is not an intraocular, but rather a similar, crossreactive extraocular or environmental antigen. This phenomenon of “antigenic mimicry” is postulated to be the initiating event of most autoimmune diseases, and we and others have described a series of molecules and epitopes from pathogens (bacteria, viruses, fungi, parasites) and some harmless nutritional molecules (bovine milk casein) that can serve as primary activating antigens for subsequent induction of uveitis (4–7). Antigenic mimicry leading to crossreactive T cell recognition is based on the manner of antigen peptide recognition by T cell receptors: the receptor molecule does not screen each amino acid side chains of a peptide but only a few amino acids, therefore many different peptides with similar amino acids at a certain position are sufficient for the activation of the receptor (**Figure 1**) (8, 9). This is a pivotal way to increase the repertoire of antigen recognition with a restricted number of receptors, which is desirable for the defense against pathogens, but less appreciated when antigens from pathogens and autoantigens are confused. Antigenic mimicry exists for T cell as well as antibody recognition (10), here we are focusing on mimicry and T cell responses.

## Mimotopes of Retinal Autoantigens

### Environmental Pathogens and Harmless Microbes

In contrast to autoantigens, infections with pathogens, like viruses or bacteria, alert cells of the innate immune system to initiate an adaptive immune response by activating B and T cells. If these pathogens accidentally provide antigen peptides with similarity to autoantigens, crossreactive immune responses can lead to an attack focused on self-antigens expressed by the host tissue. Molecules from pathogens can thus serve as mimicry molecules and activate T cells. After reactivation of these cells by crossreactive ocular antigens they recruit non-specific leukocytes causing inflammation and destruction of ocular tissue. Among these microbial mimicry candidates are proteins from microbiota like E. coli or yeast as well as pathogenic viruses like rotavirus.

In 1989 Singh et al. reported the induction of experimental autoimmune uveitis (EAU) in Lewis rats with a synthetic peptide corresponding to a sequence of E. coli protein and a similarity with 6 aa from a uveitogenic peptide of retinal S-Ag (peptide M, aa303-320). This similarity was sufficient for crossreactivity of T lymphocytes to these peptides *in vitro* (11).

A synthetic peptide from yeast Histone 3 with a sequence homology of 5 N-terminal amino acids with peptide M was also uveitogenic in rats in a dose-dependent manner and induced similar ocular damage/inflammation as the retinal autoantigen S-Ag or its Peptide M. This mimicry was confirmed by the adoptive transfer of T cells specific for the yeast histone 3 peptide that induced EAU as well (4, 5, 12).

In the follow up a series of peptides were identified with homology to retinal S-Ag, including peptides from Hepatitis B virus, Baboon virus, murine leukemia virus, murine sarcoma virus or potato proteinase IIa. They all induced signs of EAU in Lewis rats upon immunization, but in some cases very high doses (up to 2 mg/rat) were needed. These peptides, defined as pathogenic mimotopes in rats were also capable of inducing a proliferating response in PBMC isolated from S-Ag-immunized monkeys (13).

We showed that a peptide from the outer capsid protein of rotavirus (Rota), a gastrointestinal pathogen, shares amino acid homologies with the uveitogenic retinal S-antigen peptide PDSAg (aa 341-354). It is also able to induce EAU in Lewis rats after immunization and adoptive transfer of Rota-specific T cell lines (**Table 1**) (6). Rota- and PDSAg-specific rat T cell lines recognize both peptides, PDSAg and Rota, respectively. Rota-induced EAU was indistinguishable from PDSAg-induced disease, but the incidence of uveitis was reduced. Antigenic mimicry of rotavirus and ocular peptide was also shown by enhanced antibody- and T cell responses to both antigens from patients with uveitis compared to healthy individuals (6).

**Table 1:** Mimotopes of retinal autoantigen in uveitis.

Source	Amino acid sequence of peptide/epitope															Protein
Rotavirus				W	T	E	V	S	E	V	A	T	E	V		Outer capsid protein aa 591-601 (peptide Rota)
Bovine milk				S	E	E	S	A	E	V	A	T	E	E	V	Bovine αs2-casein aa 73-84 (peptide Cas)
**Retinal autoantigen**	**F**	** L**	** G**	** E**	** L**	** T**	** S**	** S**	** E**	** V**	** A**	** T**	** E**	** V**		**Human retinal S-Ag,** **peptide PDSAg aa 341-354**
HLA-B	A	L	N	E	D	L	S	S	W	T	A	A	** D**	T		HLA-B, α1 domain aa 125-138 (peptide B27PD)
**Retinal autoantigen**	** V**	** T**	** I**	** Y**	** L**	** G**	** N**									**Human retinal S-Ag** **aa 26-32**
*M. bovis* (BCG)	V	T	I	Y	L	G	N									Invasion protein aa 433-439
**Retinal autoantigen**	** V**	** G**	** T**	** P**	** A**	** E**	** Q**	** A**								**Human IRBP aa 304-311**
*M. bovis* (BCG)	V	G	T	P	A	E	V	A								MabA protein aa 231-238
**Retinal autoantigen**	** D**	** G**	** S**	** S**	** W**	** E**	** G**									**Human IRBP, peptide R14** **aa 1200-1206**
*M. bovis* (BCG)	D	G	S	S	W	D	G									Membrane acyltransferase aa 173-179
**Retinal autoantigen**	** D**	** S**	** L**	** S**	** P**	** E**	** A**									**Human CRALBP aa 129-135**
*M. bovis* (BCG)	D	S	L	S	P	E	A									Polyketide synthase aa 997-1003

S-Ag, retinal soluble antigen; IRBP, interphotoreceptor retinoid-binding protein; CRALBP, cellular retinaldehyde-binding protein, M. bovis, Mycobacterium bovis. Amino acids identical with the sequence of the retinal autoantigen are marked by grey fields.

Bacille-Calmette-Guérin (BCG), a nonpathogenic strain of Mycobacterium bovis, has been used as a vaccine against tuberculosis and as treatment for bladder carcinoma. Uveitis is a known, but rare potential side effect, although BCG does not infect the eye. We therefore postulated antigenic mimicry of BCG proteins with ocular autoantigens to explain ocular inflammation, since we could show that peripheral blood lymphocytes (PBL) from a 70 year old patient, who developed uveitis for the first time after the third cycle of BCG-treatment within 3 years for bladder carcinoma, responded to PPD (purified protein derivative of M. tuberculosis) as well as various ocular proteins and peptides with proliferation and secretion of cytokines including IFN-γ, IL-6, IL-8, TNF-α and MCP-1. T cells clones to directly test crossreactivity were not available from this patient. A data base search identified sequence homologies of several potential epitopes of M. tuberculosis proteins with retinal autoantigens like S-Ag, interphotoreceptor retinoid-binding protein (IRBP) and cellular retinal-binding-protein (cRALBP), suggesting antigenic mimicry (**Table 1**) (7).

In transgenic as well as in “classic” murine EAU models Horai et al. have demonstrated that commensal bacteria in the gut may contribute to ocular autoimmunity through a molecular mimicry mechanism. Alteration of the gut microbiota may contribute to ocular autoimmunity at several stages: (1) innate microbial stimuli, (2) loss of distinct microbiota producing anti-inflammatory stimuli, (3) presence of pathogenic bacteria disturbing the intestinal barrier and secreting inflammatory mediators, (4) and mimicry of microbiota proteins with autoantigens (14–17). Antigenic mimicry of heat shock proteins (hsp) from gut microbiota and hsp expressed by retinal ganglion cells and axons as a stress response to elevated intraocular pressure has recently been described as a potential cause of chronic progressive destruction of neuronal cells in normal tension glaucoma. The pathogenesis of chronic, low-grade autoreactive T cell responses initiated in the gut and finally acting in the eye is similar to that postulated for autoimmune uveitis (18).

In addition, antigenic mimicry between ocular antigens and antigens derived from (commensal) bacteria, yeast, viruses and parasites might lead to the activation of pathogenic autoreactive immune responses. Subsequent ocular inflammation after infection with Onchocerca volvulus has been proposed as antigenic mimicry between worm proteins and ocular autoantigens; immunization of Lewis rats with recombinant protein (Ov39) from Onchocerca volvulus caused ocular inflammation, and T cells from Ov39-immunized rats responded to an ocular protein (hr44) *in vitro*. The pathogenicity of Ov39-specific T cells was confirmed by adoptive transfer experiments (19).

### Mimicry of Food Antigens

Like the eye, the gut is a tissue with a strong ability to induce tolerance. Soluble protein antigens, for example from food, without providing PAMPs (pathogen-associated molecular pattern) to activate TLR (Toll like receptors) or inflammasomes, usually induce tolerance when they get access to cells of the immune system in submucosal tissues. This phenomenon is called “mucosal tolerance” or “oral tolerance” (20, 21). Oral tolerance is effective systemically. Regulatory T cells induced in the gut are screening the body systemically and prevent activation of effector cells to nutritional antigen. This is necessary, because food proteins are foreign molecules that might not be completely digested to the level of amino acids before being absorbed. As entire intact proteins they could induce adverse systemic immune reactions. Failure of oral tolerance induction could result in food allergies, but in general this mechanism is very reliable and therefore immune reactions to nutritional proteins are rare events, also when the food proteins are mimicking antigens from pathogens or autoantigens. Antigenic mimicry of food antigens and antigens from infectious agents has been described for antibody binding, Vodjani for example demonstrated antibody crossreactivities between Borrelia burgdorferi, Epstein-Barr-virus and rotavirus and a variety of different food antigens, likely induced by the pathogens, but without clinical consequences (22).

We had previously shown antigenic mimicry of a peptide from bovine milk casein (Cas) and peptide PDSAg from retinal S-Antigen (6) (**Table 1**). We found increased antibody responses to Cas and PDSAg in sera of patients with uveitis compared to healthy individuals, as well as increased T cell responses among PBL of such patients. However, mimicry could not be proven on the level of a single TCR recognizing both mimotopes on the same MHC molecule. Moreover, we could induce experimental uveitis in Lewis rats after immunization with peptide Cas and also transfer disease by Cas-specific T cells. The primary natural contact of the immune system with Cas, as well as with the peptide from Rotavirus (see above), would be in the gut, therefore we have tried to induce uveitis by oral antigen application together with Cholera toxin as Th1-shifting gastrointestinal adjuvant (6). In contrast to the peptides, only the casein protein or bovine milk was able to induce uveitis *via* the oral challenge. Cas-specific rat T cell lines recognize peptide PDSAg and also the peptide from Rotavirus, and Cas is recognized by PDSAg- as well as Rota-specific T cells.

Normally, a nutritional protein like bovine milk casein should not induce an adverse immune reaction, unless it is accidentally presented in an inflammatory environment, such as during a gastrointestinal infection breaking oral tolerance. On the other hand, bovine milk casein is the typical nutrition of a calf, which has no oral tolerance mechanisms and is a food allergen for human infants, probably having some characteristics to induce immune defense and effector rather than regulatory T cells in humans. Moreover, we have failed to induce oral tolerance with peptide Cas and also with peptide Rota from rotavirus to prevent PDSAg-induced uveitis, while the autoantigen PDSAg itself is a very strong oral tolerogen (6, 23).

### Self-Antigens Mimicking Autoantigens

Since antigenic mimicry is only dependent on similar antigenic peptides to induce crossreactive immune responses also the body’s own antigens can mimic each other. Self-antigens usually do not provide “danger” signals to initiate innate and immune effector responses, so they should induce tolerance. We have shown antigenic mimicry of the retinal S-Antigen peptide PDSAg (human S-Ag, aa341-352, FLGELTSSEVATEV) and an oligomorphic peptide from the first domain of HLA-B antigens (B27PD, aa125-138, ALNEDLSSWTAADT) (**Table 1**). This peptide was originally thought to be specific for the amino acid sequence of HLA-B27 and thus regarded as an explanation for the strong HLA-class I-B27-association with anterior uveitis, despite the dominant role of HLA-class II-restricted T-helper cells in uveitis (24). Later it turned out that the sequence represented by peptide B27PD is found in the first domain of most HLA-B-molecules and thus not specific for HLA-B27. The consequence was that most patients with uveitis bear at least one HLA-B molecule with the sequence of B27PD. Therefore, we hypothesized that this peptide could represent a key mimotope for most types of uveitis. In fact, lymph node cells from PDSAg-immunized Lewis rats as well as lymphocytes from patients with uveitis showed crossreactivity to both, PDSAg and B27PD, in proliferation assays *in vitro*. However, the HLA-peptide B27PD was only marginally pathogenic after immunization in rat EAU. In contrast, the HLA-peptide B27PD was highly tolerogenic when applied orally to rats prior to induction of uveitis with S-Ag or peptide PDSAg (oral tolerance).

Surprisingly, in rat EAU the oral tolerance-inducing mimotopes were recognized by γδ^+^T cell receptors and not by αβ^+^T cells that induce the disease (25). We have shown this by adoptive transfer of tolerance to PDSAg-induced EAU with CD8^+^γδ^+^T cells from rats fed with either peptide PDSAg itself or with the mimotope B27PD. Moreover, we have demonstrated the *in vitro*-proliferation of orally induced γδ^+^T cells in response to their tolerogen or the respective mimicry peptide. In this case, the αβ^+^TCR^+^ effector T cells served as antigen-presenting cells, and the proliferation of the CD8^+^TCR-γδ^+^ cells was impeded by the addition of antibodies blocking the peptide-restricted MHC class II-molecule as well as by antibodies blocking CD8 (25, 26).

These findings favored a new hypothesis for natural self-tolerance in the gut, maintained by constant shedding and processing of HLA-molecules from intestinal epithelial cells (e.g. HLA-B molecules with the sequence of B27PD) and inducing mucosal tolerance to self-HLA molecules. This tolerance might be promoted and specified by additional oral application of the peptide B27PD with crossreactivity to the retinal autoantigen, thus leading to therapeutic tolerance in uveitis (27, 28).

Peptides from any self protein, including HLA, can be presented on HLA-molecules and might serve as a constant trigger of self-tolerance for the immune system. Immunomodulatory properties of peptides derived from the sequence of HLA-molecules have also been described by others (29). Due to its high tolerogenic potency and a lack of pathogenicity the peptide B27PD was successfully used as an oral tolerogen for patients with uveitis in a phase 1 therapeutic trial (30–32). However, a following placebo-controlled phase I/II trial with this oral peptide did lack efficacy and was completed early (clinicaltrials.gov-identifier NCT01195948, September 7, 2020).

## Discussion

Low-affinity self-reactive T cells are found in the body where these autoreactive T cells are normally controlled. These pre-existing, self-reactive T cells can be activated in response to similar antigens from environmental microbes and/or food and become pathogenic under certain circumstances.

Molecular mimicry of environmental antigens in conjunction with the ability of T cells to escape immune tolerance has been suggested as a potential mechanism for the pathogenesis not only of autoimmune uveitis but also of other autoimmune diseases, including: multiple sclerosis, diabetes mellitus and spondylarthropathies (10).

Retinal autoantigens are sequestered behind the BRB (1, 2) and therefore normally invisible for naïve T cells. Since only activated T-lymphocytes can pass the BRB and enter the eye their primary activation must be extraocular.

The activation of pattern recognition receptors (PRR) by pathogens is a prerequisite for the initiation of an immune response, however, this is lacking for usually tolerizing food proteins. In that situation we proposed a concomitant infection with the presentation of the food antigen (33), leading to an erroneous bystander activation of effector T cells instead of (oral) tolerance induction. For example to orally induce EAU with milk casein we used Cholera toxin for the activation of PRR in the gut-associated lymphoid tissue (GALT) (34).

Interestingly, in addition to peripheral activation, which enables the T cells to pass the BRB, the barrier itself and the passage of the T cells through the barrier seemed to be essential for the pathogenicity of the T cells, since intravitreal injection of autoreactive T cells does not cause inflammation (35).

Autoimmune uveitis is mediated by CD4^+^Th cells which recognized peptide presented on MHC class II molecules. For an antigen recognition the minimum prerequisite is the binding of the antigen peptide to the presenting MHC molecule and the subsequent recognition of this complex by a TCR. Crossreactivity of antigen peptides could be observed either with partially overlapping consecutive amino acid sequences, identities between ocular antigens and viral or bacterial proteins (4, 5, 11–13, 36) or by discontinuous sequence homologies with amino acids at respective positions to anchor the peptide to the MHC and also to bind to the TCR (6) (**Figure 1**). The interactions between antigen peptides, presenting MHC and TCR are based on charges of amino acid side chains, hydrogen bonds and complementary structures. Even a peptide with structural homology but not sharing identical/similar amino acids can be sufficient for antigenic mimicry (10, 37), thus complicating the search and definition of mimotopes.

While Wucherpfennig and Strominger could demonstrate antigenic mimicry with similar peptides at the clonal level of T cells (38), a certain T cell might also express a second T cell receptor, one recognizing a foreign peptide and the other a self peptide (39, 40). We have shown a cross-reactivity on the T cell population level (41), but we could not prove the use of a certain single T cell receptor to crossreact with peptides PDSAg, Rota and Cas (unpublished). In that case, we might not have a single T cell receptor recognizing all three peptides, but perhaps several T cells crossreacting with two of the mimotopes, finally resulting in crossreactivity to all three peptides on the population level of T cell lines.

Most peptide mimics were defined for retinal S-Ag in animal models and in humans (5, 6, 11–13, 24, 36). We have demonstrated the proliferative response and cytokine secretion to several ocular autoantigens (IRBP, S-Ag, cRALBP) of PBL from a patient experiencing uveitis as an adverse event after BCG-treatment. Whether this rarely observed recognition of multiple retinal autoantigens is due to mimicry of multiple mycobacterial antigens and several autoantigens or just a broad autoimmune response developing over time by epitope spreading cannot be ascertained (7). Both, intramolecular as well as intermolecular epitope spreading has been described in experimental models of uveitis (42–44).

Autoreactive lymphocytes are not only found in the peripheral blood of patients with autoimmune diseases but also in healthy people (45, 46), indicating that further conditions are required for pathogenicity. We assume that in addition to the presence of autoreactive T cells the target organ itself contributes to the initiation and maintenance of uveitis. In the eye the autoantigen must be expressed and properly processed and presented by local antigen-presenting cells to reactivate immigrating T cells with a corresponding receptor followed by the recuitment of inflammatory cells that finally cause destruction of ocular tissues (18, 47).

## Author Contributions

GW: Writing the manuscript, design of the figure MD-M: writing the manuscript. All authors contributed to the article and approved the submitted version.

## Conflict of Interest

The authors declare that the research was conducted in the absence of any commercial or financial relationships that could be construed as a potential conflict of interest.

## References

[1] Streilein JW Ocular immune privilege: the eye takes a dim but practical view of immunity and inflammation. J Leukocyte Biol (2003) 74(2):179–85. 10.1189/jlb.1102574 12885934

[2] Shechter R London A Schwartz M Orchestrated leukocyte recruitment to immune-privileged sites: absolute barriers versus educational gates. Nat Rev Immunol (2013) 13(3):206–18. 10.1038/nri3391 23435332

[3] Chu XK Chan C-C Sympathetic ophthalmia: to the twenty-first century and beyond. J Ophthalmic Inflammation Infect (2013) 3(1):49–. 10.1186/1869-5760-3-49 PMC3679835 23724856

[4] Shinohara T Singh VK Tsuda M Yamaki K Abe T Suzuki S S-antigen: from gene to autoimmune uveitis. Exp Eye Res (1990) 50(6):751–7. 10.1016/0014-4835(90)90125-E 2197111

[5] Shinohara T Singh VK Yamaki K Abe T Tsuda M Suzuki S S-antigen: molecular mimicry may play a role in autoimmune uveitis. Prog Clin Biol Res (1991) 362:163–90. Issn 0361-7742. 10.1002/eji.1830241103 2003125

[6] Wildner G Diedrichs-Möhring M Autoimmune uveitis induced by molecular mimicry of peptides from rotavirus, bovine casein and retinal S-antigen. Eur J Immunol (2003) 33(9):2577–87. 10.1002/eji.200324058 12938234

[7] Garip A Diedrichs-Mohring M Thurau SR Deeg CA Wildner G Uveitis in a patient treated with Bacille-Calmette-Guerin: possible antigenic mimicry of mycobacterial and retinal antigens. Ophthalmology (2009) 116(12):2457–62.e1-2. 10.1016/j.ophtha.2009.05.021 19815288

[8] Daniel C Horvath S Allen PM A Basis for Alloreactivity: MHC Helical Residues Broaden Peptide Recognition by the TCR. Immunity (1998) 8(5):543–52. 10.1016/S1074-7613(00)80559-2 9620675

[9] Sundberg EJ Deng L Mariuzza RA TCR recognition of peptide/MHC class II complexes and superantigens. Semin Immunol (2007) 19(4):262–71. 10.1016/j.smim.2007.04.006 PMC2949352 17560120

[10] Oldstone MBA Molecular mimicry and immune-mediated diseases. FASEB J (1998) 12(13):1255–65. 10.1096/fasebj.12.13.1255 PMC7164021 9761770

[11] Singh VK Yamaki K Abe T Shinohara T Molecular mimicry between uveitopathogenic site of retinal S-antigen and Escherichia coli protein: induction of experimental autoimmune uveitis and lymphocyte cross-reaction. Cell Immunol (1989) 122(1):262–73. 10.1016/0008-8749(89)90166-4 2665946

[12] Singh VK Yamaki K Donoso LA Shinohara T Sequence homology between yeast histone H3 and uveitopathogenic site of S-antigen: lymphocyte cross-reaction and adoptive transfer of the disease. Cell Immunol (1989) 119(1):211–21. 10.1016/0008-8749(89)90237-2 2465832

[13] Singh VK Kalra HK Yamaki K Abe T Donoso LA Shinohara T Molecular mimicry between a uveitopathogenic site of S-antigen and viral peptides. Induction of experimental autoimmune uveitis in Lewis rats. J Immunol (Baltimore Md 1950) (1990) 144(4):1282–7. 1689349

[14] Horai R Zárate-Bladés CR Dillenburg-Pilla P Chen J Kielczewski JL Silver PB Microbiota-dependent activation of an autoreactive T cell receptor provokes autoimmunity in an immunologically privileged site. Immunity (2015) 43(2):343–53. 10.1016/j.immuni.2015.07.014 PMC4544742 26287682

[15] Zárate-Bladés CR Horai R Caspi RR Regulation of Autoimmunity by the Microbiome. DNA Cell Biol (2016) 35(9):455–8. 10.1089/dna.2016.3432 PMC5031118 27463238

[16] Zárate-Bladés CR Horai R Mattapallil MJ Ajami NJ Wong M Petrosino JF Gut microbiota as a source of a surrogate antigen that triggers autoimmunity in an immune privileged site. Gut Microbes (2017) 8(1):59–66. 10.1080/19490976.2016.1273996 28045579 PMC5361604

[17] Horai R Sen HN Caspi RR Commensal microbiota as a potential trigger of autoimmune uveitis. Expert Rev Clin Immunol (2017) 13(4):291–3. 10.1080/1744666X.2017.1288098 PMC5546913 28145784

[18] Chen H Cho K-S Vu THK Shen C-H Kaur M Chen G Commensal microflora-induced T cell responses mediate progressive neurodegeneration in glaucoma. Nat Commun (2018) 9(1):3209. 10.1038/s41467-018-06428-2 30097565 PMC6086830

[19] McKechnie NM Gurr W Yamada H Copland D Braun G Antigenic mimicry: Onchocerca volvulus antigen-specific T cells and ocular inflammation. Invest Ophthalmol Visual Sci (2002) 43(2):411–8. 11818385

[20] von Boehmer H Oral tolerance: is it all retinoic acid? J Exp Med (2007) 204(8):1737–9. 10.1084/jem.20071251 PMC2118658 17620364

[21] Weiner HL da Cunha AP Quintana F Wu H Oral tolerance. Immunol Rev (2011) 241(1):241–59. 10.1111/j.1600-065X.2011.01017.x PMC3296283 21488901

[22] Vodjani A Reaction of Monoclonal and Polyclonal Antibodies Made against Infectious Agents with Various Food Antigens. J Clin Cell Immunol (2015) 6:359. 10.4172/2155-9899.1000359

[23] Wildner G Diedrichs-Möhring M Autoimmune uveitis and antigenic mimicry of environmental antigens. Autoimmun Rev (2004) 3(5):383–7. 10.1016/j.autrev.2004.01.002 15288005

[24] Wildner G Thurau SR Cross-reactivity between an HLA-B27-derived peptide and a retinal autoantigen peptide: a clue to major histocompatibility complex association with autoimmune disease. Eur J Immunol (1994) 24(11):2579–85. 10.1002/eji.1830241103 7957552

[25] Wildner G Hunig T Thurau SR Orally induced, peptide-specific gamma/delta TCR+ cells suppress experimental autoimmune uveitis. Eur J Immunol (1996) 26(9):2140–8. 10.1002/eji.1830260927 8814259

[26] Wildner G Thurau SR Diedrichs-Möhring M Gamma-delta T cells as orally induced suppressor cells in rats: in vitro characterization. Ann New Y Acad Sci (2004) 1029:416–21. 10.1196/annals.1309.050 15681796

[27] Thurau SR Wildner G An HLA-peptide mimics organ-specific antigen in autoimmune uveitis: its role in pathogenesis and therapeutic induction of oral tolerance. Autoimmun Rev (2003) 2(4):171–6. 10.1016/S1568-9972(03)00011-9 12848942

[28] Mannie MD DeOca KB Bastian AG Moorman CD Tolerogenic vaccines: Targeting the antigenic and cytokine niches of FOXP3+ regulatory T cells. Cell Immunol (2020) 355:104173. 10.1016/j.cellimm.2020.104173 32712270 PMC7444458

[29] Clayberger C Krensky AM Immunosuppressive peptides corresponding to MHC class I sequences. Curr Opin Immunol (1995) 7(5):644–8. 10.1016/0952-7915(95)80071-9 8573307

[30] Thurau SR Diedrichs Mohring M Fricke H Burchardi C Wildner G Oral tolerance with an HLA-peptide mimicking retinal autoantigen as a treatment of autoimmune uveitis. Immunol Lett (1999) 68(2-3):205–12. 10.1016/S0165-2478(99)00071-1 10424422

[31] Thurau SR Diedrichs-Möhring M Fricke H Arbogast S Wildner G Molecular mimicry as a therapeutic approach for an autoimmune disease: oral treatment of uveitis-patients with an MHC-peptide crossreactive with autoantigen–first results. Immunol Lett (1997) 57(1-3):193–201. 10.1016/S0165-2478(97)00058-8 9232451

[32] Thurau SR Fricke H Burchardi C Diedrichs-Mohring M Wildner G Long-term follow-up of oral tolerance induction with HLA-peptide B27PD in patients with uveitis. Ann New Y Acad Sci (2004) 1029:408–12. 10.1196/annals.1309.048 15681794

[33] Voigt V Wikstrom ME Kezic JM Schuster IS Fleming P Makinen K Ocular antigen does not cause disease unless presented in the context of inflammation. Sci Rep (2017) 7(1):14226. 10.1038/s41598-017-14618-z 29079770 PMC5660195

[34] Wilson AD Bailey M Williams NA Stokes CR The in vitro production of cytokines by mucosal lymphocytes immunized by oral administration of keyhole limpet hemocyanin using cholera toxin as an adjuvant. Eur J Immunol (1991) 21(10):2333–9. 10.1002/eji.1830211007 1833201

[35] Mochizuki M Kuwabara T McAllister C Nussenblatt RB Gery I Adoptive transfer of experimental autoimmune uveoretinitis in rats. Immunopathogenic mechanisms and histologic features. Invest Ophthalmol Vis Sci (1985) 26(1):1–9. 3967952

[36] Singh VK Yamaki K Donoso LA Shinohara T Molecular mimicry. Yeast histone H3-induced experimental autoimmune uveitis. J Immunol (Baltimore Md 1950) (1989) 142(5):1512–7. 2645363

[37] Quaratino S Thorpe CJ Travers PJ Londei M Similar antigenic surfaces, rather than sequence homology, dictate T-cell epitope molecular mimicry. Proc Natl Acad Sci (1995) 92(22):10398. 10.1073/pnas.92.22.10398 7479792 PMC40804

[38] Wucherpfennig KW Strominger JL Molecular mimicry in T cell-mediated autoimmunity: viral peptides activate human T cell clones specific for myelin basic protein. Cell (1995) 80(5):695–705. 10.1016/0092-8674(95)90348-8 7534214 PMC7133435

[39] Schuldt NJ Binstadt BA Dual TCR T Cells: Identity Crisis or Multitaskers? J Immunol (2019) 202(3):637. 10.4049/jimmunol.1800904 30670579 PMC11112972

[40] Elliott JI Altmann DM Dual T cell receptor alpha chain T cells in autoimmunity. J Exp Med (1995) 182(4):953–9. 10.1084/jem.182.4.953 PMC2192287 7561698

[41] Mason D A very high level of crossreactivity is an essential feature of the T-cell receptor. Immunol Today (1998) 19(9):395–404. 10.1016/S0167-5699(98)01299-7 9745202

[42] Deeg CA Thurau SR Gerhards H Ehrenhofer M Wildner G Kaspers B Uveitis in horses induced by interphotoreceptor retinoid-binding protein is similar to the spontaneous disease. Eur J Immunol (2002) 32(9):2598–606. 10.1002/1521-4141(200209)32:9<2598::AID-IMMU2598>3.0.CO;2-# 12207344

[43] Boldison J Khera TK Copland DA Stimpson ML Crawford GL Dick AD A novel pathogenic RBP-3 peptide reveals epitope spreading in persistent experimental autoimmune uveoretinitis. Immunology (2015) 146(2):301–11. 10.1111/imm.12503 PMC4582971 26152845

[44] Diedrichs-Mohring M Hoffmann C Wildner G Antigen-dependent monophasic or recurrent autoimmune uveitis in rats. Int Immunol (2008) 20(3):365–74. 10.1093/intimm/dxm148 18203685

[45] Goebels N Hofstetter H Schmidt S Brunner C Wekerle H Hohlfeld R Repertoire dynamics of autoreactive T cells in multiple sclerosis patients and healthy subjects: Epitope spreading versus clonal persistence. Brain (2000) 123(3):508–18. 10.1093/brain/123.3.508 10686174

[46] Danke NA Koelle DM Yee C Beheray S Kwok WW Autoreactive T Cells in Healthy Individuals. J Immunol (2004) 172(10):5967. 10.4049/jimmunol.172.10.5967 15128778

[47] Thurau SR Mempel TR Flugel A Diedrichs Mohring M Krombach F Kawakami N The fate of autoreactive, GFP+ T cells in rat models of uveitis analyzed by intravital fluorescence microscopy and FACS. Int Immunol (2004) 16(11):1573–82. 10.1093/intimm/dxh158 15351788

